# Central Composite Design for Optimization of Mitomycin C-Loaded Quantum Dots/Chitosan Nanoparticles as Drug Nanocarrier Vectors

**DOI:** 10.3390/pharmaceutics15010209

**Published:** 2023-01-06

**Authors:** Fariza Aina Abd Manan, Nor Azah Yusof, Jaafar Abdullah, Armania Nurdin

**Affiliations:** 1 Institute of Nanoscience and Nanotechnology, Universiti Putra Malaysia, UPM Serdang, Serdang 43400, Selangor, Malaysia; 2 Department of Chemistry, Faculty of Science, Universiti Putra Malaysia, UPM Serdang, Serdang 43400, Selangor, Malaysia; 3 Department of Biomedical Sciences, Faculty of Medicine and Health Sciences, Universiti Putra Malaysia, Serdang 43400, Selangor, Malaysia

**Keywords:** Mitomycin C, drug delivery, Mn:ZnS quantum dots, chitosan, response surface methodology

## Abstract

Cancer is one of the most devastating diseases that leads to a high degree of mortality worldwide. Hence, extensive efforts have been devoted to the development of drug nanocarrier vectors as a potential new cancer treatment option. The main goal of this treatment is to deliver an anticancer medicine successfully and effectively to the patient’s cells using non-toxic nanocarriers. Here, we present a drug delivery system to emphasize the optimization of an anticancer drug-loaded formulation using Mitomycin C (MMC) encapsulated in chitosan nanocarrier conjugated with a bioimaging fluorescence probe of Mn:ZnS quantum dots (MMC@CS-Mn:ZnS). Additionally, the Response Surface Methodology (RSM), which uses a quadratic model to forecast the behaviour of the nano-drug delivery system, was used to assess the optimization of encapsulation efficiency. In this investigation, the core points of the Central Composite Design (CCD) model were used with 20 runs and 6 replications. The encapsulation efficiency (EE%) was measured using UV-Vis spectroscopy at 362 nm. The highest EE% is 55.31 ± 3.09 under the optimum parameters of incubation time (105 min), concentration of MMC (0.875 mg/mL), and concentration of nanocarriers (5.0 mg/mL). Physicochemical characterizations for the nanocarriers were accessed using a nanosizer and field-emission scanning electron microscopy (FESEM). Three independent variables for the evaluation of the encapsulation efficiency were used, in which the incubation time, concentration of MMC, concentration of nanocarriers, and correlation for each variable were studied. Furthermore, the MMC drug release efficiency was carried out in four different solution pHs of 5.5, 6.0, 6.5, 7.0, and pH 7.5, and the highest cumulative drug release of 81.44% was obtained in a pH 5.5 release medium, followed by cumulative releases of 68.55%, 50.91%, 41.57%, and 32.45% in release mediums with pH 6.0, pH 6.5, pH 7.0, and pH 7.5. Subsequently, five distinct mathematical models—pseudo-first-order, pseudo-second-order, Hixson-Crowell, Korsmeyer-Peppas, and Higuchi kinetic models—were used to fit all of the drug release data. The Korsmeyers-Peppas model was found to fit it well, highlighting its importance for the log of cumulative drug release proportional to the log of time at the equilibrium state. The correlation coefficient value (R^2^) was obtained as 0.9527, 0.9735, 0.9670, 0.9754, and 0.9639 for the drug release in pH 5.5, pH 6.0, pH 6.5, pH 7.0, and pH 7.5, respectively. Overall, from the analysis, the as-synthesized MMC nanocarrier (MMC@CS-Mn:ZnS) synergistically elucidates the underlying efficient delivery of MMC and leverages the drug loading efficiency, and all these factors have the potential for the simultaneous curbing of non-muscle invasive bladder cancer reoccurrence and progression when applied to the real-time disease treatment.

## 1. Introduction

The prognosis of non-muscle invasive bladder cancer (NMIBC) involves approximately 75% of incident cases of bladder cancer [1,2], and the frequent reoccurrence and progression may lead to more lethal muscle-invasive bladder cancer (MIBC) [3]. Subsequently, the quest for frequent surveillance episodes and continuous treatment systems became an urgent necessity to combat NMIBC reoccurrence and their progression in the human body [4]. Surgical intervention, immunotherapy, photodynamic therapy, chemotherapy, and radiotherapy are all effective treatments for patients suffering from bladder cancer. These treatment regimens are assigned based on several factors, including the type of bladder cancer, the stage of disease, the extent of metastasis, the patient’s overall health, and personal preferences [5]. Although these strategies give an excellent curative effect, they failed to prevent chemoresistance and bladder cancer progression. First, this treatment may cause harm towards the neighbouring healthy cells due to the lack of specificity in biodistribution and low concentration of drug delivered at targeted cancer cells, which leads to adverse health side effects [6]. Second, the chemotherapy efficacy is limited by drug resistance, which leads to low efficacy and insufficient delivery of drugs to cancer cells resulting in an incomplete treatment. Hence, a high dose of drug administration is required, which leads to tissue toxicity and drug resistance [7,8]. In this regard, a drug delivery system has been developed as drug resistance has been a major impediment in cancer therapy.

Drug delivery systems (DDS) have an array of prominent solutions to improve drug biocompatibility in cells and tissues, increase intracellular uptake, maintain drug stability, and facilitate drug biodistribution to the targeted cancer cells or tissues without causing any harm to the adjacent non-cancer cells, etc. This can be possible with the DDS because drug nanocarriers were specially designed to deliver drugs to specific site-targeted cells which exhibit a high affinity towards the receptors overexpressed specifically by the cancer cells rather than healthy cells [9,10]. Herein, we used targeted nanoparticles (NPs) to enhance the delivery of anticancer therapeutics to the cancerous cell with high efficacy and sustained intracellular drug levels. Recently, numerous anticancer NPs-based drugs have been clinically approved, and more are undergoing clinical trials. The main hurdle in DDS is to localize the NPs to the targeted cancerous tissue. Hence, the overexpression of receptors on the surface of cancer cells has been harnessed for the targeted delivery of therapeutics using a ligand/antibody-modified nano-drug carrier system using nanomaterials as vehicles. Recently, myriad kinds of nanomaterials have been employed with multifaceted applications in DDS, and this includes the distribution of drugs or therapeutic compounds with enhanced biodistribution, high specificity towards the cancer cells, extended systemic circulation, no/minimal toxicity to the host cells, and with non-invasive molecular imaging behaviour [11,12].

Amongst the naturally occurring mucoadhesive cationic polysaccharides, chitosan is a natural biopolymer scaffold that exhibits numerous functional groups for the binding of targeting ligands by surface modification [13]. Chitosan nanoparticles (CSNPs) possess excellent properties as a drug nanocarrier vector prior to their characteristic feature, including biodegradability, biocompatibility, mucoadhesive ability, and low toxicity, which accelerate the drug nanomaterial penetration across the mucosal surfaces of the cell [14]. CSNPs can be implemented in a varied array of research areas, such as biosensors, biocatalysis, biomedicine, pharmaceuticals, and bioimaging, etc. In this research, CSNPs were synthesized using an ionotropic gelation approach, a simple physical cross-linking technique. This approach involves the ionic interaction between two opposite charges of positively charged amino groups from chitosan and the negatively charged phosphate groups of sodium tripolyphosphate (TPP). The synthesized CSNPs from this approach were asserted to be non-toxic and hindered the possibility of drug degradation. In addition, the positively charged CSNPs exhibit mucoadhesive properties that will enhance the nanomaterial permeation and simultaneously facilitate the opening of epithelial tight junction [15]. In particular, the CSNPs hybrid formulations of nano DDS have evoked substantial progress and fascinating results in the theragnostic of cancer cell diagnosis and simultaneous treatment. For the reason these hybrid nano DDS are amenable to physiochemical modification in terms of particle size, shape, and surface modification, they are versatile for a wide range of applications in pharmacology and biomedicine. Other than that, the solubility, drug retention, immunogenicity, and biodistribution could be improved, and all these factors can curb drug resistance towards cancer cells [16].

Semiconductor nanocrystals, also known as quantum dots (QDs), are extensively employed as bioimaging agents rather than fluorophores and organic dyes, and they changed the paradigm of fluorescence in the past decades. Traditionally, various kinds of fluorophores have been employed in bioimaging applications, such as phycobiliproteins [17], fluoresceins [18], rhodamines [19], and porphyrins [20,21], etc.; however, they are susceptible to photobleaching, low signal intensity, and spectral overlapping. Due to their multiplex emission with single light stimulation, little overlap, and strong resistance to the photobleaching effect, QDs have been introduced to overcome these drawbacks [22]. It has been reported in previous literature that the advantage of the successful conjugation of CSNPs with QDs opens a new horizon as a drug nanocarrier, including low toxicity, biocompatibility, and enhanced surface functionality [22,23,24,25].

In this work, a new approach was developed to encapsulate an anticancer drug, mitomycin-C (MMC), within the CS-Mn:ZnS nanocarrier to form MMC@CS-Mn:ZnS. The main aim of the present work was to attain an optimum nanocarrier design with maximum encapsulation efficiency. The design of experiment (DoE) using the Response Surface Methodology (RSM) was used to study the relationship between three independent variables and responses and establish the interdependent relations between the variables by sequential experimentation [25]. The analysis was conducted by a three-factor central composite design (CCD) approach, which involves the factors of the incubation time, concentration of MMC, and concentration of nanocarriers, respectful to the encapsulation efficiency response. This optimization is crucial as MMC is a water-soluble drug and can decelerate the cell penetration and internalization dynamics of extracellular cells due to the lipophilic nature of cell membranes. For the drug release kinetic studies, the drug release profiling data have been plotted based on five different mathematical models. Hence, our study presents a facile approach for the synthesis of a biocompatible and water-dispersible MMC@CS-Mn:ZnS nanocarrier as a smart excipient for drug delivery with remarkable anticancer therapeutic efficiency. To the best of our knowledge, this represents a novel approach in the emerging research on CS conjugated with Mn:ZnS assembly for therapeutic applications, particularly in non-muscle invasive bladder cancer.

## 2. Materials and Methods

### 2.1. Materials

Mitomycin C (Cat. No. 3258, CAS No. 50-07-7, C_15_H_18_N_4_O_5_, M_w_ = 334.33 g·mol^−1^) drugs of analytical grades were obtained from Bio-Techne, Tocris Bioscience (Bristol, UK). Chitosan (CAS No. 9012-76-4, low molecular weight, M_w_ = 50,000–190,000 Da, 75–85% degree of acetylation) and sodium tripolyphosphate (CAS No. 7758-29-4, Na_5_O_10_P_3_, M_w_ = 367.86 g·mol^−1^) were procured from Sigma-Aldrich (St. Louis, MO, USA). Manganese (II) sulphate monohydrate (CAS No. 10034-96-5, MnSO_4_⋅H_2_O, 99%, M_w_ = 169.02 gmol^−1^, 99%), zinc acetate dihydrate (CAS No. 5970-45-6, Zn(CH_3_COO)_2_·2H_2_O, M_w_ = 219.51 g·mol^−1^, 99.5%) and sodium sulphide nonahydrate (CAS No. 1313-82-2, Na_2_S·9H_2_O, M_w_ = 240.18 g·mol^−1^, 98%) were purchased from R&M Marketing (Essex, UK). Other reagents were of analytical grade and used without further purification. All aqueous solutions were prepared using ultrapure water of resistivity (18.2 MΩ·cm) and purified using a Thermo Scientific water purification system.

### 2.2. Methods

#### 2.2.1. Response Surface Methodology

Response Surface Methodology (RSM) is a mathematical approach that merges the experimental design and statistical technique for empirical model building. RSM is a less laborious approach which is widely used to forecast an optimal experimental condition and clarify the effect of several independent variables towards the response. Conventionally, the one variable at a time method was used prior to its simplicity. However, the correlation effects that involve several variables need numerous experimental works to predict the optimum condition [26,27]. In this work, RSM based on Central Composite Design (CCD) was implemented to study the correlation of three variables (incubation time, concentration of MMC, and concentration of nanocarriers) towards the encapsulation efficiency (response). Each independent variable in the Central Composite Design can be set to a low level (−1), a central level (0), or a high level (+1). To conduct the RSM/CCD, a statistical package software was used (Design Expert 11.0, Stat Ease Inc., Minneapolis, MN, USA). The correlation of the levels of several input variables (or predictors) and the output variable (or response) was estimated using a statistical approach, Analysis of variance (ANOVA). In these analyses, the confidence level was set at 99%.

##### Central Composite Design

Central composite design (CCD) exhibits two-level factorials (−1 and +1), augmented with two types of points (axial or star points and central points), which are crucial factors for the estimation of quadratic effects. All of the factors in the axial point are set to zero. However, there is an exception for the value coded with ± α, in which the value can be in the range of 1 to $\sqrt{k}$ (k = factors). In this surface response model, the value represented by the axial points that exhibit these extreme values differentiates between the axial points and the factorial design. [28,29].

##### Optimization of Formulations by Factorial Design

The correlation of three independent factors was studied, which are the incubation time (A), concentration of MMC (B), and concentration of nanocarriers (C), corresponding to the encapsulation efficiency as the response variable. The expression for CCD consists of the number of experiments, N = $2^{n}$ (cube portion) + $n_{c}$ (Star portion) + Cp, where Cp is the central point, as expressed in (Equation 1). Table 1 summarises the experimental settings for these parameters, which were derived from CCD. A total of 20 experimental runs, including 8 factorial points, 6 axial points, and 6 central points, were used to optimise these three independent components, which were then evaluated using Equation (2):

(1) $$N=2^{n}+n_{c}+Cp$$

(2) $$N=2^{3}+2\left(3\right)+6=20$$

A second-order polynomial model was used prior to its flexibility and accuracy in the determination of the curvature and the interactions; hence, it suits the optimization process and infers non-trivial phenomena [28], as expressed by Equation (3):

(3) $$Y=\beta_{0}+\sum_{i=1}^{k}\beta_{i}\chi_{i}+\sum_{i=1}^{k}\beta_{ii}\chi_{i}^{2}+\;\sum_{i=1}^{k-1}\sum_{j>1}^{k}\beta_{ij}\chi_{i}\chi_{j}+\varepsilon$$

where *Y* is an objective to optimize the response, *β_0_* is the constant coefficient, *β_i_* is the linear coefficient, *β_ii_* is the quadratic coefficient, and *β_ij_* is the interaction coefficient, while *x_i_* and *x_j_* represent the coded values of the independent factors, and ε is the random error component that is determined by fitting the model to the data [30,31].

Response surface modelling was used to determine the best conditions for the three independent variables in order to maximise encapsulation efficiency. Table 1 depicts the regression interaction between three actual independent variables coded as (−1, 0, +1) and subdivided into three levels (low, middle, and high).

##### Statistical Analysis

ANOVA was used to calculate various statistical parameters, including the degree of freedom (DF), the significance of the proposed model, the sequential sum of squares (Seq SS), the modified sum of squares (Adj SS), the contribution of each model coefficient and each factor, the predicted residual error sum of squares (PRESS), the standard deviation (S), the coefficient of determination (R^2^), the adjusted R^2^ (R^2^_Adj_), and the predicted R^2^ (R^2^_pred_). Data were provided as the mean ± standard deviation, and an ANOVA with *p* < 0.0001 was used to assess the significance difference between the parameters.

#### 2.2.2. Preparation of MMC@CS-Mn:ZnS Nanocarriers and Encapsulation Efficiency

##### Preparation of MMC@CS-Mn:ZnS Nanocarriers

By adapting the previously mentioned procedure, Mn:ZnS QDs, a fluorescent probe, were synthesized [12]. Briefly, three different aqueous precursors were prepared, namely, 0.15 M MnSO_4_⋅H_2_O, 0.10 M Zn(CH_3_COO)_2_⋅2H_2_O, and 0.10 M Na_2_S·xH_2_O. Furthermore, 3 mL of MnSO_4_⋅H_2_O was mixed with 20 mL of Zn(CH_3_COO)_2_⋅2H_2_O and dropped wisely under a continuous 40 kHz-operating ultrasonic bath. N_2_ gas was used to de-aerate the solution for 15 min to eradicate unwanted dissolved gases that may interrupt the synthesis process. Next, 20 mL of Na_2_S·xH_2_O was added wisely into the mixture with continuous N_2_ purging and stirring at 200 rpm. The resultant precursor of Mn-doped ZnS (Mn:ZnS) was treated with microwave irradiation at 1000 W for 60 s in a sealed Teflon vessel at 120 °C to produce high purity samples. The suspension was then subjected to UV irradiation for 20 min.

The MMC was then loaded onto the CS-Mn:ZnS nanocarrier using the ionic gelation process with minor modifications. In brief, 5 mg of CS powder was dissolved in 1 mL of 1.0% (*v/v*) acetic acid solution to make 5 mg/mL of CS solution. Furthermore, 2.5 µL of Mn:ZnS was added dropwise into 250 µL of CS solution under continuous stirring. In the meantime, 1 mg/mL of MMC was also prepared. Following that, a homogenous solution was prepared by sonicating 250 µL of CS solution and 250 µL of MMC in a ratio of 1:1 (*v/v*). A 1:100 (*v/v*) volume ratio of Tween-20 to CS solution was then added and dispersed at a concentration of 2% (*v/v*) in deionized water to avoid particle aggregation. Tween-20 is used as a stabilizing and capping agent in this work. It interacts with water molecules through its hydrophilic characteristics, speeding up the interaction of NPs with the aqueous medium.

Tween-20 stabilizes NPs despite its safe qualities; it was introduced because NPs production occurs at this stage. CS NPs were constructed spontaneously after the addition of 100 µL TPP (10 mg/mL) under continuous stirring at 200 rpm with an optimum ratio of TPP to CS of 1.0:2.5 (*v/v*). The mixture was then centrifuged three times for 10 min at 12,000 rpm and the supernatant was discarded. Lastly, the MMC@CS-Mn:ZnS nanocarrier pellet was then freeze-dried overnight before further usage.

##### Encapsulation Efficiency

The encapsulated MMC was extracted by dispersing 5.0 mg of each lyophilized nanocarrier formulation in a mixture of 1.0 mL of methanol and 0.5% HCl (*v/v*). Sonication was continuously applied to dissolve the nanocarriers completely and release 100% of the drug that was entrapped inside. Lastly, the nanoparticles were centrifuged at 12,000 rpm for 10 min, and the supernatant was collected for UV-Vis Spectroscopy analysis at 362 nm. The concentration of MMC was extrapolated from the MMC standard calibration plot. Furthermore, the encapsulation efficiency (EE) was calculated using the following Equation (4):

(4) $$\mathrm{EE}\;\left(\%\right)=\frac{W_{i}}{W_{t}}\times 100$$

where *W_i_* is the weight of the drug in the nanocarrier and *W_t_* is the weight of the drug in the system.

#### 2.2.3. Drug Release Studies

10.0 mg of the synthesized nanocarriers were initially dispersed in 10.0 mL of phosphate-buffered saline solution (PBS) with a pH of 5.5 to 7.5. The solution was then stirred at 27 °C and 100 rpm for 480 min. At predetermined time intervals, 1 mL of the solution was collected to estimate the concentration of mitomycin C released using UV-Vis spectroscopy at 362 nm. The remaining solution was topped up with the same concentration of the fresh medium.

#### 2.2.4. Characterization

The surface morphology of the synthesized nanocarriers was obtained using Field-emission scanning electron microscopy (FESEM, JSM-7500F JEOL, Tokyo, Japan). The photophysical characteristics of the nanocarriers and the amount of drug loading and release at λ = 362 nm were analysed by Multiskan GO Microplate Spectrophotometer (Thermo Fischer Scientific, Waltham, MA, USA). The hydrodynamic particle size and surface charge of the nanocarriers were assessed by dynamic light scattering (DLS) studies using a particle size analyzer (Nano Series Nano- ZS, Malvern Panalytical Ltd., Malvern, UK).

## 3. Results and Discussion

### 3.1. Drug delivery System

**Figure 1:**
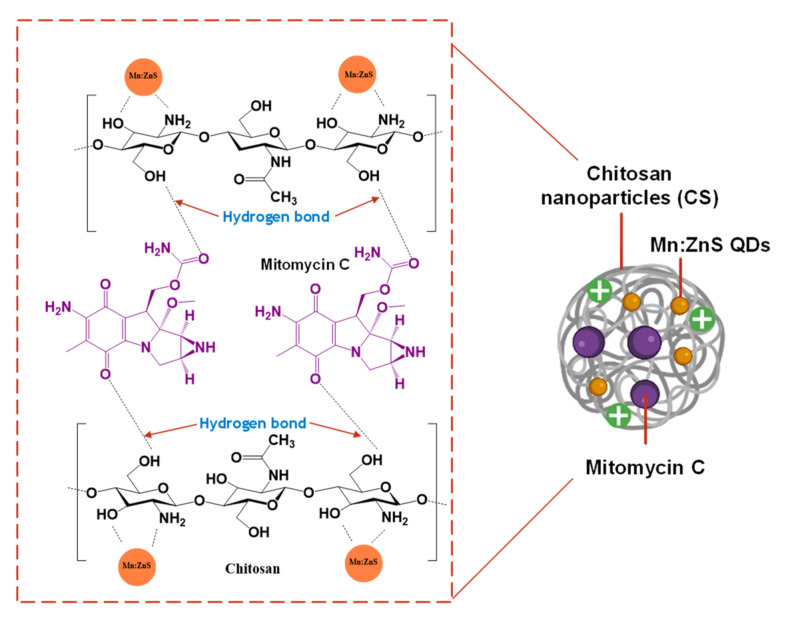
Mechanistic approach of the MMC@CS-Mn:ZnS drug excipient.

Herein, we designed a drug delivery system based on chitosan nanoparticles (CS). The mechanistic approach of the MMC@CS-Mn:ZnS drug excipient is presented as a scheme in Figure 1. Chitosan exhibits the amino group (-NH_2_) and hydroxyl group (-OH) and acts as the chelating agent that will effectively coordinate the Mn^2+^ and Zn^2+^ of the quantum dots. The presence of the functional groups is crucial to maintaining the stability and particle size of the quantum dots [32]. In this drug delivery system, the MMC loaded into entangled CS with the fluorescence probe of Mn:ZnS quantum dots (MMC@CS-Mn:ZnS) was formulated via a facile method of the ionic gelation method by the addition of the crosslinking agent, sodium tripolyphosphate (TPP) [33,34]. This formulation has been reported in our previous work [12] and further discussed in Section 2.2.2. Previously, the highest encapsulation efficiency (EE%) with the value of 60.31 ± 0.49 was obtained with an MMC concentration of 1.00 mg/mL (with two fixed variables: the incubation time = 300 min and the concentration of nanocarriers = 10.00 mg/mL). In this current work, the incubation time and the concentration of the nanocarrier were successfully studied and optimized. The highest EE% is 55.31 ± 3.09, with an MMC concentration of 0.875 mg/mL, an incubation time of 105 min, and the concentration of the nanocarrier is 5.00 mg/mL. Although the EE% slightly decreases after the optimization of those three variables, the incubation time was reduced three-fold from 300 min to 105 min, so the drug encapsulation can be achieved in a shorter time, with lower concentrations of MMC and nanocarrier. The encapsulation efficiency of different types of payloads in the chitosan-based nanocarriers has been tabulated in Table 2.

### 3.2. Building of Regression Model RSM

Encapsulation efficiency was evaluated at the centre point of design space with optimum values (incubation time = 105 min; concentration of MMC = 0.875 mg/mL; concentration of nanocarriers = 5.0 mg/mL), which give the highest response value of 55.31 ± 3.10% (Table 3).

By utilizing a multiple regression analysis on the obtained experimental data, a second-order polynomial regression equation was obtained in terms of coded factors and is presented below (Equation (5)):

Y = 51.9211 + 9.39058 A + 0.62575 B + 5.47933 C + 2.59125 AB + 0.99125 AC + 0.42375 BC + −16.015 A^2^ + −7.695 B^2^ + −8.69 C^2^ (5)

where A is the incubation time, B is the concentration of MMC, and C is the concentration of nanocarriers, whereas Y represents the encapsulation efficiency in the drug delivery system. The positive sign in front of the terms indicates a synergistic effect, while the negative sign indicates an antagonistic effect [34].

Table 4 clearly tabulates the analysis of variance (ANOVA) for encapsulation efficiency, as well as the correlation of three independent variables model terms (A, B, C) and their interaction on the selected response (Y). In summary, the F-value regression of the quadratic model is 601.1335, with a small *p*-value (<0.0001) and the F-value for lack of fit is 0.517011, indicating an insignificant value that is related to pure error [31].

The quadratic model terms—A, C, AB, AC, A^2^, B^2^, and C^2^ exhibit *p*-values less than 0.05, as summarized in Table 4. The coefficient of determination (R^2^) is a crucial factor to identify the adequacy of a regression model. In this study, the R^2^ = 0.9980, implying that 99% of the variation in the encapsulation efficiency can be explained by the independent variables and only 1% can be explained by the model. The R^2^_pred_ = 0.9907 is in good agreement with the R^2^_adj_ = 0.9962. The adequate precision indicates the signal-to-noise ratio, with a value of 66.2991, in which a value greater than 4 is adequate. Meanwhile, this model exhibits a low coefficient of variation (C.V. %) with a value of 2.61, implying that the experimental data exhibits high precision and good reliability. The prediction error sum of squares (PRESS) measures how well the model predicts, and a low PRESS (32.45) result indicates a favourable model.

### 3.3. Diagnostics

**Figure 2:**
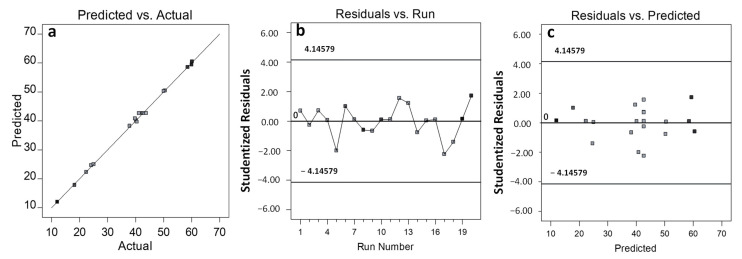
(**a**) Predicted versus actual experimental data, (**b**) studentized residual response versus the run number, and (**c**) studentized residual response versus predicted response.

The experimental data versus predicted values for encapsulation efficiency in Figure 2a represent a good correlation and a well-fitted model. Figure 2b shows the normal probability plot of studentized residuals against the run number to determine any persistent errors that might occur in the data analysis. The result obtained shows a randomly scattered plot without any definite patterns or trends, which validates that no persistent error was found. The studentized residuals versus the predicted data plot, as shown in Figure 2c, verifies that the data was consistently distributed by the mean point of the surface response and, hence, validated that this model can be classified as a satisfactory model with zero persistent error.

### 3.4. Correlation of Significant Factors Involved in the Encapsulation Efficiency

Two-dimensional (2D) and three-dimensional (3D) response surfaces are provided as graphical representations of the regression equation [39] in order to better comprehend the interaction between the variables, as further discussed using Equation (5) in Section 3.1.

**Figure 3:**
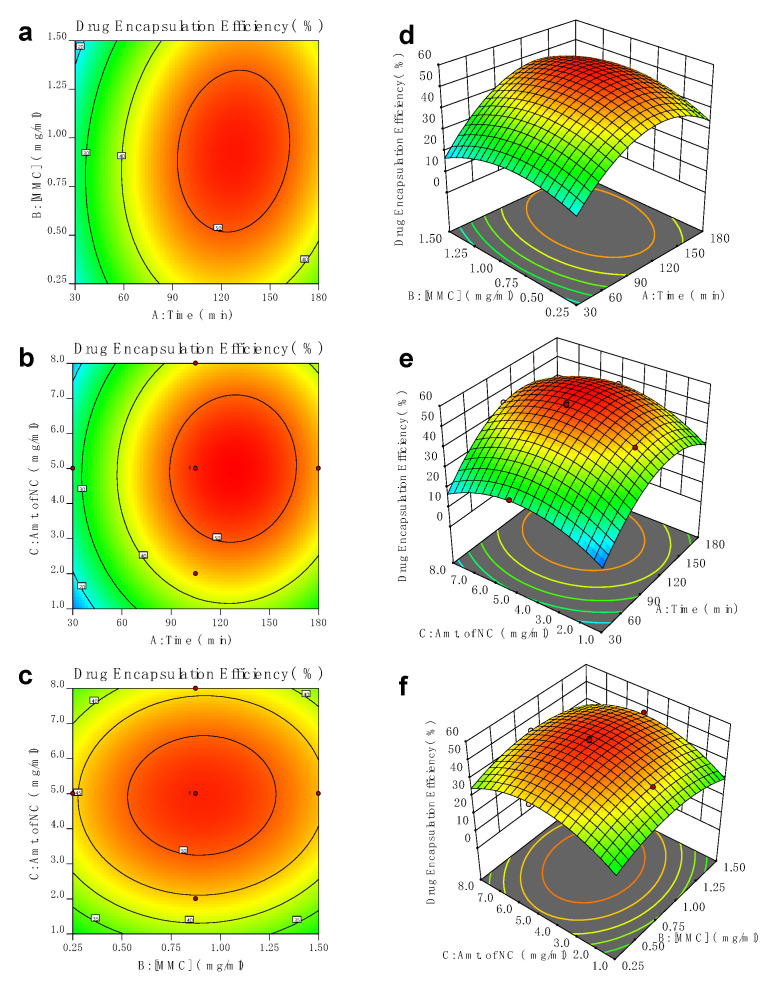
Plots in 2D contour space as a function of (**a**) incubation time (min), (**b**) concentration of MMC, and (**c**) concentration of nanocarriers and 3D surface as a function of (**d**) incubation time (min), (**e**) concentration of MMC, and (**f**) concentration of nanocarriers.

When the other variables are fixed to 0, each contour curve in Figure 3 represents an endless number of possibilities between two significant variables. The surface was contained within the contour diagram’s smallest ellipse for the 2D diagram, which signifies the highest anticipated value for encapsulation efficiency. The interaction between incubation time and the (A)- concentration of MMC (B) gives the maximum encapsulation efficiency at an incubation time of 105 min, with the MMC concentration reaching 0.875 mg/mL, as shown in Figure 3a. Figure 3b shows the interaction between the incubation time and the (A) concentration of nanocomposites (C), which maximizes the encapsulation efficiency at an incubation time of 105 min and a concentration of nanocarriers of 5.0 mg/mL. The interaction of drug (B)-concentration of nanocarriers (C) was clearly shown in (Figure 3c), in which the encapsulation efficiency exhibits the highest percentage at a concentration of MMC at 0.875 mg/mL, coupled with the concentration of nanocarriers of 5.0 mg/mL. All the three-dimensional (3D) response surface are clearly represented in Figure 3d–f.

### 3.5. Validation of Model and Optimization of Encapsulation Efficiency

Three sets of tests produced by Design Expert software to validate the predictive model by comparing the predicted and experimental values. The reaction variables, such as the incubation time, concentration of MMC, and concentration of nanocarriers, were set in a range between low and high levels coded as −1 and +1, as tabulated in Table 5. The optimization process was carried out by inserting the maximum encapsulation efficiency as the desired goal.

#### Encapsulation Efficiency (EE %)

A set of numerical optimization menus were successfully generated based on experimental data, and three sets of experimental data were chosen, as shown in Table 6, to envisage the optimum reaction parameters with optimum values and achieve model validity. The results give a low residual standard error (RSE) of less than 2%, which suggests that the model is valid with a high encapsulation efficiency of up to 98%. The optimized encapsulation efficiency generated from RSM was compared with the experimental value of absorbance calculated from UV-Vis Spectroscopy at a wavelength of 362 nm. The data revealed that maximum encapsulation efficiency was obtained when the highest capability of the MMC was encapsulated into the chitosan matrix. The EE% of MMC in CS was found to be 54.316 ± 0.007 for the experimental data and 55.13 ± 0.18 for the predicted data. In addition, the encapsulation efficiency was aided by the freeze-dried approach, in which the drug nanocarrier pellets were allowed to lyophilize, concentrated while freezing, and the MMC were closely packed and fused in the chitosan-based nanocarrier and encapsulated efficiently [40].

### 3.6. Physicochemical Characterization of MMC@CS-Mn:ZnS Drug Nanocarrier

**Figure 4:**
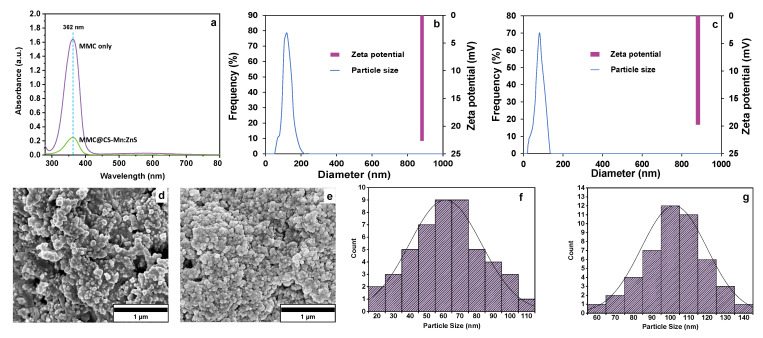
Physico-chemical characterization of the biocomposite scaffolds: (**a**) UV-Vis spectroscopy for MMC and MMC@CS-Mn:ZnS Particle size distribution and zeta potential for (**b**) CS and (**c**) MMC@CS-ZnS. FESEM images for (**d**) CS and (**e**) MMC@CS-Mn:ZnS. Histogram for particle size distribution for (**f**) CS and (**g**) MMC@CS-Mn:ZnS.

Figure 4a shows the UV-vis spectra of MMC and MMC@CS-Mn:ZnS nanocarriers, where the absorbance peak was observed at a wavelength of 362 nm. The decrease in the absorbance peak is observed for the MMC@CS-Mn:ZnS nanocarrier due to the interaction of MMC and the CS-Mn:ZnS nanocarrier [41], indicating that MMC was successfully loaded to MMC@CS-Mn:ZnS.

The particle size and zeta potential of the nanocarriers were also studied, as the size and charge of nanocarriers can affect their cellular uptake and their distribution in organs. For the particle size, 80.45 ± 1.06 nm and 105.43 ± 0.88 nm were obtained for CS (Figure 4b) and MMC@CS-Mn:ZnS nanocarrier (Figure 4c), respectively. The incorporation of any substances or drugs into chitosan nanoparticles could increase the particle size, as reported in previous reports [35]. Similarly, in this study, the MMC@CS-Mn:ZnS nanocarrier showed greater particle size due to the incorporation of drugs.

Zeta potentials for CS and MMC@CS-Mn:ZnS nanocarriers are 19.69 ± 1.02 mV and 22.63 ± 1.50 mV, respectively. Only a slight shifting in the zeta potential was observed between CS and MMC@CS-Mn:ZnS. This observation suggested that the encapsulation of MMC did not affect the surface charge. Therefore, we can conclude that the MMC was not conjugated onto the nanocarrier but was successfully encapsulated. It is anticipated that the positively charged nanocarriers could convey a better interaction with the negatively charged mucosal membrane, accelerating the delivery of MMC on the targeted cancer cells and simultaneously increasing cellular uptake [35].

FESEM images were photographs at 100,000 magnifications, as shown in Figure 4d,e, while the histogram of particle size distribution obtained from FESEM and analyzed using ImageJ is shown in Figure 4f,g. The FESEM images for CS and MMC@CS-Mn:ZnS show spherical shapes with a slightly rough surface texture, with which this morphology could facilitate an efficient drug delivery process, possibly due to the higher surface area provided by the nanocarriers [35]. The average particle size for CS and MMC@CS-Mn:ZnS range from 60–70 nm and 100–110 nm, respectively. CS exhibits smaller particle sizes because it has not been encapsulated with the Mn:ZnS fluorescence probe and MMC drug.

### 3.7. In Vitro Release Profile of MMC

In vitro release profile of MMC was conducted using four different media (phosphate buffer solution) with a pH ranging from 5.5 to 7.5 and in a period of 480 min. This study was carried out to investigate the mechanisms and interactions inside the nanocarrier with respect to the pH response. The highest cumulative drug with a value of 81.44% was obtained in the pH 5.5 release medium, followed by the cumulative release of 68.55%, 50.91%, 41.57% and 32.45% in release medium with pH 6.0, pH 6.5, pH 7.0 and pH7.5. The swelling behaviour of chitosan in acidic conditions facilitates the drug release from the chitosan nanocarrier. At the highest pH (pH 7.5), the swelling behaviour is unfavourable, resulting in the slower release of the drug.

Basically, there are two key phases to the drug release mechanism: the burst release phase in the first phase and the sustain release phase in the second phase [42]. In the first stage, the drug releases rapidly with a burst effect, suggesting that the release of drugs on the surface of the nanocarriers by adsorption and attachment [36] can easily detach and leach out into the release medium. Meanwhile, the second phase, or sustain release phase, involves the drug release from the core structure of the CS-Mn:ZnS nanocarriers [43,44] in the MMC of Mn:ZnS.

**Figure 5:**
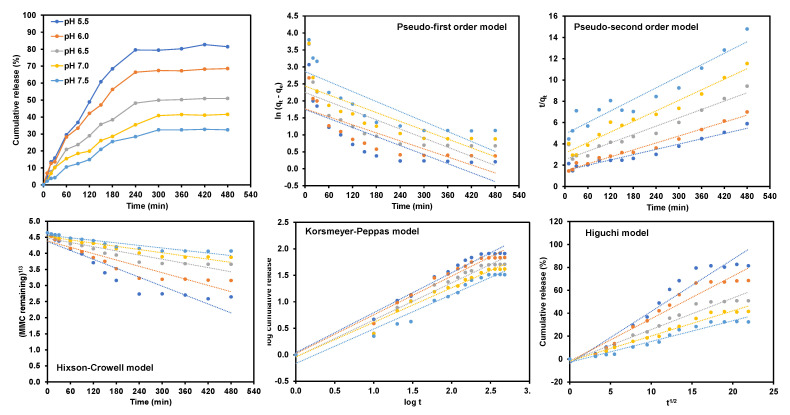
Drug release data fitted with five different mathematical models at different release medium with pHs 5.5, 6.0, 6.5, 7.0, and 7.5.

Five models were used to study the drug release profile data, which are pseudo-first-order, pseudo-second-order, Hixson-Crowell, Korsmeyer-Peppas, and Higuchi pharmacokinetic, which is shown in Figure 5. The kinetic parameters are summarized in Table 7.

With the highest correlation coefficient values (R^2^) of 0.9527, 0.9735, 0.9670, 0.9754, and 0.9639 for the drug release in pH 5.5, pH 6.0, pH 6.5, pH 7.0, and pH 7.5, respectively, the release of MMC was prominently fitted with the Korsmeyer-Peppas model and revealed a more significant prospective towards the correlation of the cumulative drug release, directly proportional with the function square root of time at equilibrium.

## 4. Conclusions

In this work, the optimization of the encapsulation efficiency was successfully evaluated using RSM based on Central Composite Design (CCD). The statistical analysis implies that the correlation of the incubation time, concentration of MMC, and concentration of nanocarriers with the encapsulation efficiency were statistically significant. The optimum parameters from RSM were found to be incubation time (105 min), concentration of MMC (0.875 mg/mL), and concentration of nanocarriers (5.0 mg/mL). This was in good agreement with the experimental data. From the analysis, the drug release data for the formulated MMC@CS-Mn:ZnS nanocarrier were fitted at four different release mediums: pH 5.5, pH 6.0, pH 6.5, pH 7.0, and pH 7.5, respectively. The highest cumulative drug with a value of 81.44% was obtained in the pH 5.5 release medium, followed by the cumulative release of 68.55%, 50.91%, 41.57% and 32.45% in release medium with pH 6.0, pH 6.5, pH 7.0 and pH7.5. The release profiling data were well fitted to the Korsmeyer–Peppas release model in explaining the mode of release by studying the relationship between the log of cumulative drug releases versus the log of times at equilibrium. These models exhibit the highest value of regression coefficients of 0.9527, 0.9735, 0.9670, 0.9754, and 0.9639 for drug release in pH 5.5, pH 6.0, pH 6.5, pH 7.0 and pH 7.5. In conclusion, the encapsulation efficiency for developed DDS is directly proportional to the drug release kinetics, which increases the encapsulation efficiency and, hence, increases the drug release profiling data. As a result, MMC@ CS-Mn:ZnS nanocarriers may be considered as an effective drug delivery strategy for bladder cancer that is non-muscle invasive, however additional in vivo research is required for future study. On that basis, an experimental investigation in vivo can be suggested to learn more about the security, penetration ability, and bioactivity of the created nanocarriers.

## Figures and Tables

**Table 1:** Experimental range and independent variable level.

Variable/Factor	Factor	Unit	Actual			Coded Level			Mean	Standard Deviation
			Low	Middle	High	Low	Middle	High		
Incubation time	A	min	30	105	180	−1	0	1	105	54.41
Conc. of MMC	B	mg/mL	0.25	0.875	1.50	−1	0	1	0.875	0.45
Conc. of nanocarrier	C	mg/mL	2.0	5.0	8.0	−1	0	1	5.00	2.18

**Table 2:** Chitosan-based nanocarriers encapsulation efficiency from previous works.

Chitosan-Based Nanocarriers	Synthesis Method	Payloads	Encapsulation Efficiency (%)	References
Chitosan nanoparticles	Ionic gelation method	L-ascorbic acid (LAA) Thymoquinone (TQ)	LAA (22.8 ± 3.2) TQ (35.6 ± 3.6)	[35]
Chitosan nanoparticles	Ionic gelation method	Hexaconazole Dazomet	Hexaconazole (65.3 ± 4.5) Dazomet (68.9 ± 3.5)	[36]
Selenium nanoparticles encapsulated by alginate-chitosan	Crosslinking/in situ reduction method	Vibrio Cholerae lipopolysaccharide LPS (nanovaccine)	62	[37]
Histidine-grafted chitosan-lipoic acid NPs (HCSL-NPs)	Single emulsion solvent evaporation method.	Paclitaxel	86.54 ± 3.51	[38]
Chitosan conjugated with Mn doped ZnS (CS-Mn:ZnS) quantum dots	Ionic gelation method	Mitomycin C	55.31 ± 3.09	This work

**Table 3:** Central composite design (CCD) for the encapsulation efficiency using RSM.

Run	Actual Independent Variable			Encapsulation Efficiency (EE) Response (%)	
	Incubation Time (min)	Conc. of MMC (mg/mL)	Conc. of Nanocarrier (mg/mL)	Experimental	Predicted
1	105	0.875	5.0	54.13	54.26
2	105	0.875	5.0	55.31	54.26
3	105	0.875	5.0	52.22	54.26
4	180	1.500	5.0	33.22	33.02
5	105	0.875	5.0	46.80	46.85
6	30	1.500	5.0	10.09	10.59
7	30	0.250	5.0	14.88	14.26
8	180	0.875	5.0	47.21	47.01
9	105	0.250	5.0	43.29	43.93
10	180	1.500	5.0	35.31	35.93
11	105	0.875	5.0	53.30	54.26
12	105	0.875	5.0	54.48	54.26
13	105	0.875	8.0	46.77	46.71
14	180	0.250	8.0	28.01	27.52
15	30	0.875	5.0	27.39	27.57
16	105	1.500	5.0	46.95	46.30
17	105	0.875	5.0	54.13	54.26
18	30	1.500	8.0	8.08	7.81
19	30	0.250	8.0	10.88	11.08
20	180	0.250	2.0	24.75	25.02

**Table 4:** ANOVA table for the optimization of encapsulation efficiency using CCD.

Source	Sum of Squares	Degree of Freedom	Mean	F-Value	*p*-Value (Prob > F)	Significance
Model	4946.56	9	549.6177	601.1335	<0.0001	significant
A-Incubation time	774.2902	1	774.2902	846.8646	<0.0001	significant
B-Conc. of MMC	3.438115	1	3.438115	3.76037	<0.0001	significant
C-Conc. of nanocarrier	114.7601	1	114.7601	125.5166	<0.0001	significant
AB	53.71661	1	53.71661	58.75148	<0.0001	significant
AC	7.860612	1	7.860612	8.597389	<0.0001	significant
BC	1.436512	1	1.436512	1.571157	<0.0001	significant
A²	705.3206	1	705.3206	771.4305	<0.0001	significant
B²	162.8358	1	162.8358	178.0985	<0.0001	significant
C²	207.6693	1	207.6693	227.1342	<0.0001	significant
Residual	9.143023	10	0.914302			
Lack of Fit	3.116023	5	0.623205	0.517011	0.7568	not significant
Pure Error	6.027	5	1.2054			
Cor Total	4955.703	19				
PRESS	32.45		R^2^	0.9980		
Std. Dev.	0.9562		Adjusted R^2^ (R^2^_adj)_)	0.9962		
Mean	36.58		Predicted R^2^ (R^2^_pred_)	0.9907		
C.V. %	2.61		Adeq. Precision	66.2991		

**Table 5:** Constraints applied for optimization.

Factor	Goal	Limit	
		Lower	Upper
A: Incubation time	Must be in the range of 30 to 180	30	180
B: Conc.of MMC	Must be in the range of 0.25 to 1.50	0.25	1.50
C: Conc. of nanocarrier	Must be in the range of 2.0 to 8.0	2.0	8.0
Encapsulation efficiency	Maximum encapsulation efficiency	7.68	54.30

**Table 6:** The optimal combination factors for the predicted and experimental values.

Run	Incubation Time (min)	Conc. of MMC (mg/mL)	Conc. of Nanocarrier (mg/mL)	Encapsulation Efficiency (%)		
				Experimental	Predicted	RSE (%)
35	128.77	0.943	5.081	54.331	54.93	1.09
36	128.3	0.971	5.020	54.316	55.13	1.48
37	127.52	0.918	5.055	54.324	55.29	1.75

**Table 7:** Kinetic parameter for drug release from MMC@CS-Mn:ZnS nanocarrier fitted to various pharmacokinetics model.

	Correlation Coefficient, R^2^				
Release Medium pH	Pseudo-First Order	Pseudo-Second Order	Hixson-Crowell	Korsmeyer-Peppas	Higuchi
5.5	0.6074	0.9306	0.8704	0.9527	0.9368
6.0	0.6540	0.9556	0.8613	0.9735	0.9455
6.5	0.5956	0.9432	0.8674	0.9670	0.9566
7.0	0.6515	0.9608	0.9309	0.9754	0.9729
7.5	0.6927	0.9113	0.8841	0.9639	0.9548

## Data Availability

Data can be provided upon request.

## References

[1] Ho P., Moran G.W., Wang V., Li G., Virk R.K., McKiernan J.M., Anderson C.B. (2022). The effect of tumor grade heterogeneity on recurrence in non-muscle invasive bladder cancer Tag edEn. Urol. Oncol. Semin. Orig. Investig..

[2] Chou R., Dana T. (2010). Screening adults for bladder cancer: A review of the evidence for the US preventive services task force. Ann. Intern. Med..

[3] Sylvester R.J., Rodríguez O., Hernández V., Turturica D., Bauerová L., Bruins H.M., Bründl J., van der Kwast T.H., Brisuda A., Rubio-Briones J. (2020). European Association of Urology (EAU) Prognostic Factor Risk Groups for Non-muscle-invasive Bladder Cancer (NMIBC) Incorporating the WHO 2004/2016 and WHO 1973 Classification Systems for Grade: An Update from the EAU NMIBC Guidelines Panel. Eur. Urol..

[4] Galesloot T.E., Grotenhuis A.J., Kolev D., Aben K.K., Bryan R.T., Catto J.W.F., Cheng K.K., Conroy S., Dyrskjøt L., Fleshner N.E. (2021). Genome-wide Meta-analysis Identifies Novel Genes Associated with Recurrence and Progression in Non—muscle-invasive Bladder Cancer. Eur. Urol. Oncol..

[5] Jain P., Kathuria H., Momin M. (2021). Clinical therapies and nano drug delivery systems for urinary bladder cancer. Pharmacol. Ther..

[6] Tang L., Li J., Zhao Q., Pan T., Zhong H., Wang W. (2021). Advanced and Innovative Nano-Systems for Anticancer Targeted Drug Delivery. Pharmaceutics.

[7] Sultan M.H., Moni S.S., Madkhali O.A. (2022). Characterization of cisplatin-Loaded chitosan nanoparticles and rituximab - linked surfaces as target - specific injectable nano-formulations for combating cancer. Sci. Rep..

[8] Meng Q., Zhong S., Xu L., Wang J., Zhang Z., Gao Y., Cui X. (2022). Review on design strategies and considerations of polysaccharide-based smart drug delivery systems for cancer therapy. Carbohydr. Polym..

[9] Babu A., Amreddy N., Muralidharan R., Pathuri G., Gali H., Chen A., Zhao Y.D., Munshi A. (2017). Chemodrug delivery using nanoparticle for lung cancer therapy. Sci. Rep..

[10] Hernandez-Giottonini K.Y., Rodrıguez-Cordova R.J., Gutierrez-Valenzuela C.A., Peñuñuri-Miranda O., Zavala-Rivera P., Guerrero-Germán P., Lucero-Acuña A. (2020). PLGA nanoparticle preparations by emulsi fi cation and nanoprecipitation techniques: Effects of formulation parameters. RSC Adv..

[11] Kukkar D., Kukkar P., Kumar V., Hong J., Kim K., Deep A. (2021). Recent advances in nanoscale materials for antibody-based cancer theranostics. Biosens. Bioelectron..

[12] Aina F., Manan A., Yusof N.A., Abdullah J., Mohammad F., Nurdin A. (2021). Drug Release Profiles of Mitomycin C Encapsulated Quantum Dots—Chitosan Nanocarrier System for the Possible Treatment of Non-Muscle Invasive Bladder Cancer. Pharmaceutics.

[13] Zhang X., Yang X., Ji J., Liu A., Zhai G. (2016). Tumor targeting strategies for chitosan-based nanoparticles. Colloids Surf. B Biointerfaces.

[14] Siddharth S., Nayak A., Nayak D., Bindhani B.K. (2017). Chitosan-Dextran sulfate coated doxorubicin loaded PLGA-PVA- nanoparticles caused apoptosis in doxorubicin resistance breast cancer cells through induction of DNA damage. Sci. Rep..

[15] Aman R.M., Zaghloul R.A., Dahhan M.S. (2021). El Formulation, optimization and characterization of allantoin-loaded chitosan nanoparticles to alleviate ethanol-induced gastric ulcer: In-vitro and in-vivo studies. Sci. Rep..

[16] Yao Y., Zhou Y., Liu L., Xu Y., Chen Q., Wang Y. (2020). Nanoparticle-Based Drug Delivery in Cancer Therapy and Its Role in Overcoming Drug Resistance. Front. Mol. Biosci..

[17] Scheer H., Yang X., Zhao K. (2015). Biliproteins and Their Applications in Bioimaging PUB. Procedia Chem..

[18] Jiao S., Wang X., Sun Y., Zhang L., Sun W., Sun Y., Wang X., Ma P., Song D. (2018). A novel fluorescein-coumarin-based fluorescent probe for fluoride ions and its applications in imaging of living cells and zebrafish in vivo. Sens. Actuators B. Chem..

[19] Zhang F., Di Y., Li Y., Qi Q., Qian J., Fu X., Xu B., Tian W., Near-infrared F.R. (2017). Highly efficient Far Red/Near-Infrared fluorophores with aggregation-induced emission for bioimaging. Dye. Pigment..

[20] Yang L., Li H., Liu D., Su H., Wang K., Liu G., Luo X. (2019). Organic small molecular nanoparticles based on self-assembly of amphiphilic fl uoroporphyrins for photodynamic and photothermal synergistic cancer therapy. Colloids Surf. B Biointerfaces.

[21] Wu F., Chen L., Yue L., Wang K., Cheng K., Chen J., Luo X., Zhang T. (2019). Small-Molecule Porphyrin-Based Organic Nanoparticles with Remarkable Photothermal Conversion Efficiency for in Vivo Photoacoustic Imaging and Photothermal Therapy Small-Molecule Nanoparticles with Remarkable Photothermal Conversion Efficiency for in Vivo Photoacoustic Imaging and Photothermal Therapy. ACS Appl. Mater. Interfaces.

[22] Chinnathambi S., Shirahata N. (2019). Recent advances on fluorescent biomarkers of near-infrared quantum dots for in vitro and in vivo imaging. Sci. Technol. Adv. Mater..

[23] Janus Ł., Piątkowski M., Radwan-Pragłowska J., Bogdał D., Matysek D. (2019). Chitosan-Based Carbon Quantum Dots for Biomedical Applications: Synthesis and Characterization. Nanomaterials.

[24] Sepahi H., Pourmadadi M., Moradi A., Yazdian F., Omidi M. (2020). Chitosan/carbon quantum dot/aptamer complex as a potential anticancer drug delivery system towards the release of 5-fluorouracil. Int. J. Biol. Macromol..

[25] Debnath S., Parashar K., Pillay K. (2017). Ultrasound assisted adsorptive removal of hazardous dye Safranin O from aqueous solution using crosslinked graphene oxide-chitosan (GO-CH) composite and optimization by response surface methodology (RSM) approach. Carbohydr. Polym..

[26] Jindal R. (2021). RSM-CCD optimized microwave assisted synthesis of chitosan and sodium alginate based nanocomposite containing inclusion complexes of β-cyclodextrin and amlodipine besylate for sustained drug delivery systems GO. J. Drug Deliv. Sci. Technol..

[27] Muthukumaran C., Kanmani B.R., Sharmila G., Kumar N.M., Shanmugaprakash M. (2018). Carboxymethylation of pectin: Optimization, characterization and in-vitro drug release studies. Carbohydr. Polym..

[28] Tavares M., Santos J., Viegas R., Palma J., Viegas F., Bentley B., Testa F., De Carvalho M., Vit M., Chorilli M. (2021). Design of experiments (DoE) to develop and to optimize nanoparticles as drug delivery systems. Eur. J. Pharm. Biopharm..

[29] Fathiyah S., Mohamad S., Said F.M., Abdul M.S. (2020). Application of experimental designs and response surface methods in screening and optimization of reverse micellar extraction. Crit. Rev. Biotechnol..

[30] Lee R. (2019). Statistical Design of Experiments for Screening and Optimization. Chem. Ing. Tech..

[31] Rosly N.Z., Abdullah A.H., Kamarudin M.A., Ashari S.E., Ainliah S., Ahmad A. (2021). Adsorption of Methylene Blue Dye by Calix [6] Arene-Modified Lead Sulphide (Pbs ): Optimisation Using Response Surface Methodology. Int. J. Environ. Res. Public Health.

[32] Nasser H., El-bery H.M., Metwally A.A. (2019). Synthesis of CdS-modified chitosan quantum dots for the drug delivery of Sesamol. Carbohydr. Polym..

[33] Sultana T., Van Hai H., Park M., Lee S., Lee B. (2020). Controlled release of Mitomycin C from modified cellulose based thermo-gel prevents post-operative de novo peritoneal adhesion. Carbohydr. Polym..

[34] Chen Y.W., Lee H.V., Bee S., Hamid A. (2017). Investigation of optimal conditions for production of highly crystalline nanocellulose with increased yield via novel Cr (III) -catalyzed hydrolysis: Response surface methodology. Carbohydr. Polym..

[35] Othman N., Md. Jamil S.N.A., Masarudin M.J., Abdullah L.C., Daik R., Sarman N.S. (2020). l-Ascorbic Acid and Thymoquinone Dual-Loaded Palmitoyl-Chitosan Nanoparticles: Improved Preparation Method, Encapsulation and Release Efficiency. Processes.

[36] Maluin F.N., Hussein Z., Yusof A. (2019). Enhanced fungicidal efficacy on Ganoderma boninense by simultaneous co-delivery of hexaconazole and dazomet from their chitosan nanoparticles. RSC Adv..

[37] Bagheri-josheghani S., Bakhshi B. (2022). Formulation of selenium nanoparticles encapsulated by alginate-chitosan for controlled delivery of Vibrio Cholerae LPS: A novel delivery system candidate for nanovaccine. Int. J. Biol. Macromol..

[38] Chakraborty A., Roy G., Swami B., Bhaskar S. (2023). Tumor targeted delivery of mycobacterial adjuvant encapsulated chitosan nanoparticles showed potential anti-cancer activity and immune cell activation in tumor microenvironment. Int. Immunopharmacol..

[39] Mangili I., Lasagni M., Huang K., Isayev A.I. (2015). Modeling and optimization of ultrasonic devulcanization using the response surface methodology based on central composite face-centered design. Chemom. Intell. Lab. Syst..

[40] Arafa M.G., Ayoub B.M. (2017). DOE Optimization of Nano-based Carrier of Pregabalin as Hydrogel: New Therapeutic & Chemometric Approaches for Controlled Drug Delivery Systems. Sci. Rep..

[41] Bakhshpour M., Yavuz H., Denizli A. (2018). Controlled release of mitomycin C from PHEMAH—Cu (II) cryogel membranes. Artif. Cells Nanomed. Biotechnol..

[42] Janeth C., Rivas M., Tarhini M., Badri W., Miladi K., Greige-gerges H., Agha Q., Arturo S., Rodríguez G., Álvarez R. (2017). Nanoprecipitation process: From encapsulation to drug delivery. Int. J. Pharm..

[43] Wang Y., Tan Y. (2016). Enhanced drug loading capacity of 10-hydroxycamptothecin-loaded nanoparticles prepared by two-step nanoprecipitation method. J. Drug Deliv. Sci. Technol..

[44] Basu T., Pal B., Singh S. (2018). Hollow chitosan nanocomposite as drug carrier system for controlled delivery of ramipril. Chem. Phys. Lett..

